# Treatment group-specific inferences in Phase III Randomized Oncology Trials

**DOI:** 10.2340/1651-226X.2025.42663

**Published:** 2025-03-24

**Authors:** Alexander D. Sherry, Adina H. Passy, Joseph Abi Jaoude, Timothy A. Lin, Ramez Kouzy, Pavlos Msaouel, Ethan B. Ludmir

**Affiliations:** a Department of Radiation Oncology, Division of Radiation Oncology, The University of Texas MD Anderson Cancer Center, Houston, TX, USA; b Department of Radiation Oncology, Stanford University, Stanford, CA, USA; c Department of Genitourinary Medical Oncology, Division of Cancer Medicine, The University of Texas MD Anderson Cancer Center, Houston, TX, USA; d Department of Translational Molecular Pathology, Division of Pathology and Laboratory medicine, The University of Texas MD Anderson Cancer Center, Houston, TX, USA; e Department of Gastrointestinal Radiation Oncology, Division of Radiation Oncology, The University of Texas MD Anderson Cancer Center, Houston, TX, USA; f Department of Biostatistics, The University of Texas MD Anderson Cancer Center, Houston, TX, USA

**Keywords:** Random sampling, convenience samples, estimation, uncertainty, confidence intervals, generalizability

## Abstract

**Background:**

Estimation of comparative treatment effects between randomized groups is well-supported in randomized trials. By contrast, treatment group-specific inferences are challenging, as patients are selectively chosen for enrollment, and such inferences are formally discouraged by the CONSORT guidelines. The present study is the first-large scale assessment of the proportion of phase III oncology trials that present treatment group-specific inferences.

**Methods:**

Published phase III randomized oncology trials were screened from ClinicalTrials.gov. Treatment group-specific inferences were defined by the presence of 95% CI or standard error for treatment-specific outcomes.

**Results:**

A total of 774 phase III trials enrolling 568,080 patients were included. Treatment group-specific inferences were present in 58% of trials (446 of 774), and appeared to be increasing over time (adjusted odds ratio for the publication year, 1.11; 95% CI, 1.06 to 1.17; *p* < 0.0001). Of the remaining 328 trials, 49 (6%) described group-specific outcomes with measures of variability, such as interquartile range, and 279 (36%) provided point estimates only (e.g., median) for group outcomes.

**Interpretation:**

The majority of published phase III oncology trials present treatment group-specific inferences. However, this inference lacks statistical support, as patients are not randomly sampled from the underlying population, and conflicts with CONSORT guidelines. While ongoing methodological efforts to improve the transportability of treatment group-specific inferences are promising, conventional attempts to generalize treatment-specific outcomes from randomized trials may be misleading. Instead of inference, treatment group-specific outcomes should be described using measures of variability.

## Introduction

Randomized clinical trials (RCTs) randomly allocate patients to different treatments. Because the random allocation procedure determines the treatment assignment, factors that influence decision-making in clinical practice, such as age, comorbidities, and performance status, no longer confound the treatment selection at baseline. Thus, random allocation provides a strong statistical basis to allow robust inferences of comparative treatment effects, i.e., the differences in outcomes between different treatments. Inferential quantities include standard errors and confidence intervals. Inferential statistics for estimating comparative treatment effects may be robustly computed in RCTs for comparative metrics such as hazard ratios (HR), risk ratios, odds ratios, absolutely risk reduction, or survival differences [1–3]. In short, the purpose of RCTs is to compare treatments [4].

When considering initiation of a new treatment, patients and oncologists are also often interested in understanding the approximate expected median or mean survival time following that specific treatment. However, obtaining this information from RCTs is exceedingly challenging. While estimations of comparative treatment effects are well supported, treatment group-specific inferences cannot be reliably made from RCTs using conventional statistics that are not adjusted for sample selection biases. This statistical challenge exists because RCTs intentionally avoid enrollments of representative patients through the use of specific inclusion and exclusion criteria that reduce patient heterogeneity [5]. Furthermore, the eligibility criteria of RCTs define which patients are not included in the RCT, but they do not by themselves guarantee that the covariate space they defined will be representatively filled by the patients enrolled in the RCT [6]. While investigators have tight control over the treatment allocation algorithm in RCTs, far less control is typically available over the sampling process. Typically, medical RCTs enroll convenience samples of participants who are enrolled sequentially over time, have logistical access to the study, meet predefined eligibility requirements, and are willing to provide informed consent. The use of such convenience samples results in a non-systematic process that does not guarantee equal enrollment chances for each subject in any identifiable target population [7]. Therefore, outputs from the convenience sample lack validity to generalize well to any target population, and additional assumptions need to be made beyond RCT design to transport inferences to target populations. Accordingly, RCT methodologists have called to abolish the presentation of inferential quantities such as standard errors and confidence intervals for treatment group-specific outcomes such as median or mean survival [8]. This is also reflected directly in the CONSORT guidelines [9]. Instead, statistics describing the variability of the sample, such as the standard deviation or the interquartile range, should be used to characterize treatment group-specific outcomes.

Despite this well-described important limitation on RCT inference, we have anecdotally observed oncology RCTs that present treatment group-specific inferences without accounting for sample selection biases. To our knowledge, no previous studies have empirically evaluated for the prevalence of this misrepresentation at scale. The purpose of this meta-epidemiological study was to determine the proportion of phase III oncology RCTs that present treatment group-specific inferences without formally acknowledging or accounting for sample selection bias.

## Methods

Institutional review board approval was not needed for this study, as all data for this meta-epidemiological study were publicly available. Clinical trials were identified in ClinicalTrials.gov and screened using previously reported criteria to include only phase III RCTs testing interventional strategies [10]. Trials were excluded if the primary endpoint comparative treatment effect estimates had not been published in a peer-reviewed manuscript (Supplementary Figure S1).

Primary full-length trial publications were reviewed, and data on the following outcomes were recorded in a standardized database. Treatment group-specific inferences were defined as the reporting of inferential statistics for treatment-specific outcomes, such as 95% CI or standard errors of the mean or median survival times. RCTs that alternatively described the variability of each treatment group’s outcomes were noted.

Multivariable binary logistic regressions were used to calculate adjusted odds ratios (aORs) and 95% CIs for the associations of trial-level covariates and treatment group-specific inferences. Confounders for each multivariable model were determined using directed acyclic graphs (Supplementary Figure S2) [11]. All testing was 2-sided, and the significance level was defined by *p* < 0.05. Analysis was completed using SAS v9.4 (Cary, NC), and plots were made in Prism v10 (GraphPad, La Jolla, CA) or R v4.3.2.

## Results

We screened 1877 trials from ClinicalTrials.gov. Of these, 774 RCTs enrolling 568,080 patients met eligibility criteria (Supplementary Figure S1). Included RCTs were published from 2002 through 2020. Most studied systemic therapy (80%, 621 of 774 RCTs), evaluated patients with metastatic solid tumors (54%, 417 of 774 RCTs), and/or were funded by industry (78%, 600 of 774 RCTs) (Table 1). As expected, all RCTs relied on selective inclusion and exclusion criteria for creation of the trial sample (i.e. convenience sampling).

**Table 1:** Characteristics of 774 eligible trials.

Characteristic	Total	Treatment group-specific inference	
		Yes (446 trials)	No (328 trials)
Cancer stage, *n* (%)			
Solid M0	205 (26)	89 (20)	116 (35)
Solid M1	417 (54)	271 (61)	146 (45)
Hematologic	152 (20)	86 (19)	66 (20)
Cancer type, *n* (%)			
Breast	146 (19)	72 (16)	74 (23)
Gastrointestinal	97 (13)	67 (15)	30 (9)
Genitourinary	91 (12)	52 (12)	39 (12)
Hematologic	152 (20)	86 (19)	66 (20)
Thoracic	110 (14)	80 (18)	30 (9)
Other^a^	178 (23)	89 (20)	89 (27)
Treatment type, *n* (%)			
Systemic therapy	621 (80)	392 (88)	229 (70)
Local therapy	26 (3)	14 (3)	12 (4)
Supportive care	127 (16)	40 (9)	87 (27)
Cooperative group–led, *n* (%)	226 (29)	114 (26)	112 (34)
Industry-funded, *n* (%)	600 (78)	372 (83)	228 (70)
Number of enrolled patients, median (IQR)	501 (280–808)	530 (335–792)	430 (218–848)
Publication date, median (IQR)	2014 (2012–2017)	2015 (2013–2017)	2014 (2011–2016)

IQR: interquartile range; M0: nonmetastatic tumors; M1: metastatic tumors.

a Other cancer types: central nervous system, endocrine, gynecologic, head and neck, multiple, pediatric, sarcoma, and skin.

**Figure 1:**
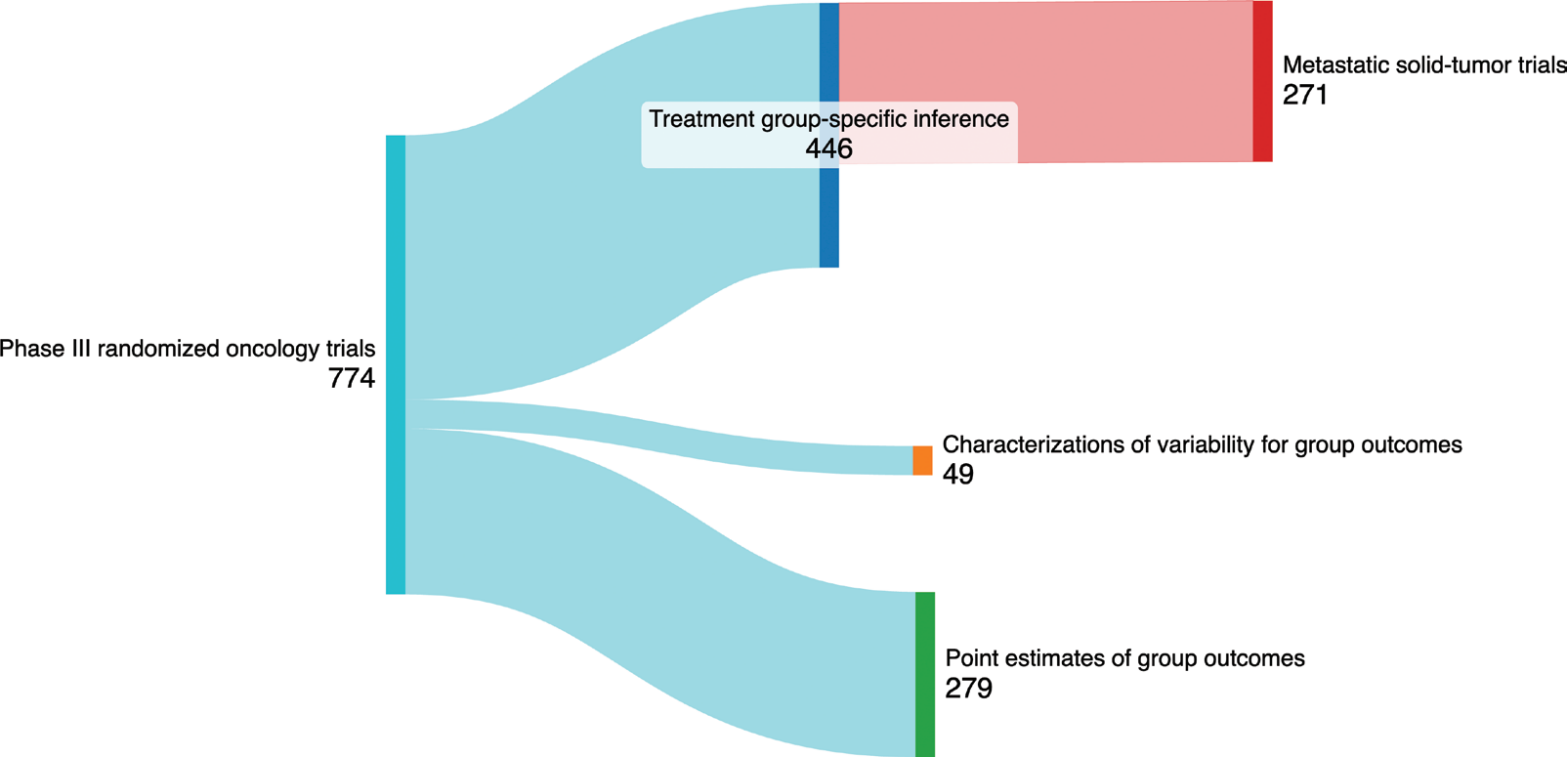
Incidence of phase III oncology RCTs presenting treatment group-specific inference.

Most RCTs (58%, 446 of 774 trials) presented treatment group-specific inferences (Figure 1). Of those, 248 RCTs included treatment group-specific inferences in the abstract. Of the remaining 328 RCTs that did not present treatment group-specific inferences, 49 (6%) RCTs described treatment group-specific outcomes with measures of variability, such as interquartile range, and 279 (36%) RCTs provided point estimates only for group outcomes, such as median survival.

**Figure 2:**
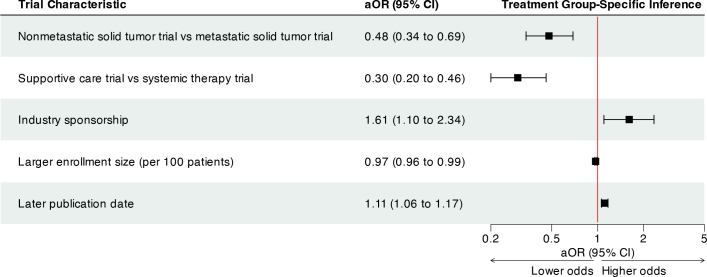
A forest plot showing the results of multivariable logistic regressions evaluating the associations of trial-level covariates and treatment group-specific inference. The whiskers represent the 95% CI of the odds ratio. The red line shows an OR of 1. aOR: adjusted odds ratio.

On univariable analysis, metastatic, solid-tumor RCTs; trials investigating systemic therapies; trials funded by industry; enrollment size; and more recently published RCTs were associated with increased odds of presenting treatment group-specific inferences (Supplementary Table S1). After adjustment for potential confounding, the presentation of treatment group-specific inferences appeared to increase over time (aOR for publication date as the covariate, 1.11; 95% CI, 1.06 to 1.17; *p* < 0.0001) (Figure 2). Trials studying supportive care seem to be less likely to present treatment group-specific inference compared with trials investigating systemic therapy (aOR, 0.30; 95% CI, 0.20 to 0.46; *p* < 0.0001). Similarly, RCTs studying nonmetastatic solid tumors were less likely to present treatment group-specific inferences than were RCTs studying metastatic solid tumors (aOR, 0.48; 95% CI, 0.34 to 0.69; *p* < 0.0001). Industry sponsorship was also associated with increased odds of presenting treatment group-specific inference (aOR, 1.61; 95% CI, 1.10 to 2.34; *p* = 0.01).

## Discussion

Despite the universal use of selective enrollment strategies without accounting for sampling selection biases, most published phase III oncology RCTs present treatment group-specific inferences. Although there is clearly an interest in understanding how treatments perform in broader populations beyond the enrolled trial patients, as evidenced by the growing use of treatment group-specific inferences over time, such efforts to generalize may mislead both patients and oncologists because RCTs do not enroll samples that are representative of a definable population. Rather than treatment group-specific inferences, RCTs should prioritize efforts on estimating comparative treatment effects, which is statistically well-supported by the random allocation procedure.

Random treatment allocation facilitates the estimation of comparative treatment effects. By contrast, random sampling facilitates treatment group-specific inferences [3, 8, 12]. Random sampling is a common technique in non-medical fields, such as political polling or computer science, and randomly draws participants from the population [12, 13]. By removing selection bias in the enrollment of participants, random sampling procedures provide robust statistical license for treatment group-specific inferences [14]. While the concept of selection bias in the enrollment of RCT participants and the random allocation procedure are both familiar to oncologists, random sampling is uncommon in medicine at large, and its specific inferential value is not often taught. This may partly explain why treatment group-specific inferences were so prevalent in our study. Furthermore, in the present study, multiple trial-level factors were associated with increased odds of treatment group-specific inferences, such as trials studying systemic therapy for metastatic solid-tumors. This may be related to an increased interest in this RCT subset for generalizing the outcomes of certain patients, or an expectation for these inferences in this subfield. It could also be a product of other factors, such as the editorial policies of journals that typically publish these RCTs. Alternatively, it may also be the case that authors wish to provide measures of outcomes more interpretable to readers than the HR from Cox regression models. In such cases, alternative inferential effect estimates that are still comparative in nature would be more appropriate to report, such as estimated absolute risk reduction, estimated restricted mean survival time differences, or even most simply the estimated differences in median survival. RCTs that do not compare group outcomes must be discouraged, as the purpose of randomization is to compare outcomes, and not estimate treatment group-specific effects [4].

The divergence between outcomes of samples in RCTs compared with samples more representative of the population have been increasingly studied. For example, a study from the Netherlands suggested that efficacy of first-line pembrolizumab for metastatic non-small cell lung cancer, in practice, appears to be significantly less than that observed on RCTs [15]. In this study, the 95% CI reported in the RCT for the median survival after pembrolizumab (18.3 months to not reached) did not even encompass the median estimate from the more representative sample (15.8 months) [15]. Notable examples of attempts to infer treatment group-specific effects from RCTs are also commonplace. For example, the 2024 National Comprehensive Cancer Network guidelines support fluorouracil/leucovorin plus oxaliplatin and irinotecan as preferred first-line therapy for locally advanced pancreatic cancer in part based on an estimated median overall survival of 24.2 months (95% CI, 21.7–26.8) obtained from a systematic review pooling multiple studies utilizing convenience samples [16, 17]. In another example, the NAPOLI-3 trial casually compares the progression-free survival outcomes from its NALIRIFOX arm vs the FOLFIRINOX arm of PRODIGE 4/ACCORD 11 in its primary publication [18]. Although there is considerable importance and interest in understanding treatment group-specific effects, these inferences are unsupported by the underlying trial design of conventional RCTs and are at best unproductive, but more likely, misleading.

Although random sampling may facilitate treatment-group-specific inferences, it is often not possible or not pragmatic to reliably identify a means by which to randomly sample populations in medicine. Studies of national databases or registries are limited by selection biases for enrolling patients on the registry, although such biases can be less prominent than the comparatively-striking selection biases of RCTs. Outcomes highly specific to RCTs, such as RECIST-defined progression, may be even more challenging to estimate from registries, as RECIST measures may not be routinely collected as standard of care, or there may be scenarios where patients are followed differently on or off trial. Importantly, there are multiple promising ongoing efforts to improve the generalizability of RCTs and transportability of treatment group-specific inference [12, 19–22]. These include pragmatic trials enrolling more representative samples as well as causal inference frameworks, although each is associated with important limitations in the absence of true random sampling [23]. This same limitation of phase III RCTs also applies to other trial designs that use convenience samples, such as single-arm phase II trials. In single-arm phase II trials, trialists should explicitly acknowledge this limitation, and strive to ensure their enrolled population is homogenous enough to require the fewest assumptions for generalizability to target populations.

Our study has several important limitations. Only primary endpoint analyses in the primary manuscript publication for RCTs registered on ClinicalTrials.gov were evaluated. We did not evaluate phase II trials or trials outside the field of oncology. Transporting comparative treatment effect estimations from RCTs to populations not included or well-represented in those RCTs also remains an important but challenging endeavor in the translation of RCT evidence to practice.

In summary, despite the lack of underlying statistical support, most phase III oncology RCTs report treatment group-specific inferences. These inferences may mislead patients and oncologists who are interested in understanding overall outcomes of specific treatment groups in populations of interest. Ongoing research in statistical methodology aims to facilitate the estimation of treatment group-specific inferences from convenience samples and improve generalizability. [12, 20, 24, 25]. These measures will require proper specification of the sampling procedure and target population of interest, as well as the collection and integration of data from that population. In the absence of these novel methods, routine inference of treatment group-specific effects from RCTs should be avoided.

## Supplementary Material

Treatment group-specific inferences in Phase III Randomized Oncology Trials

## Data Availability

Research data are stored in an institutional repository and will be shared upon reasonable request to the corresponding author.
