# Supplementary material for: Treatment group-specific inferences in Phase III Randomized Oncology Trials
Source: Acta Oncol. 2025 Mar 24;64:42663. doi: 10.2340/1651-226X.2025.42663 (PMC11959831; doi:10.2340/1651-226X.2025.42663)
Supplement: Treatment group-specific inferences in Phase III Randomized Oncology Trials [file AO-64-42663-s1.pdf]

SUPPLEMENTARY MATERIALS

Sherry AD, Passy AH, Abi Jaoude J, et al. Treatment-Group Specific Inference in Phase III Randomized Oncology Trials

Figure S1. Flowchart depicting the trial screening and selection process.

Figure S2. Directed acyclic graph used to identify confounders for multivariable regression.

Table S1. Univariable and multivariable logistic regressions.

Figure S1. Flowchart depicting the trial screening and selection process.

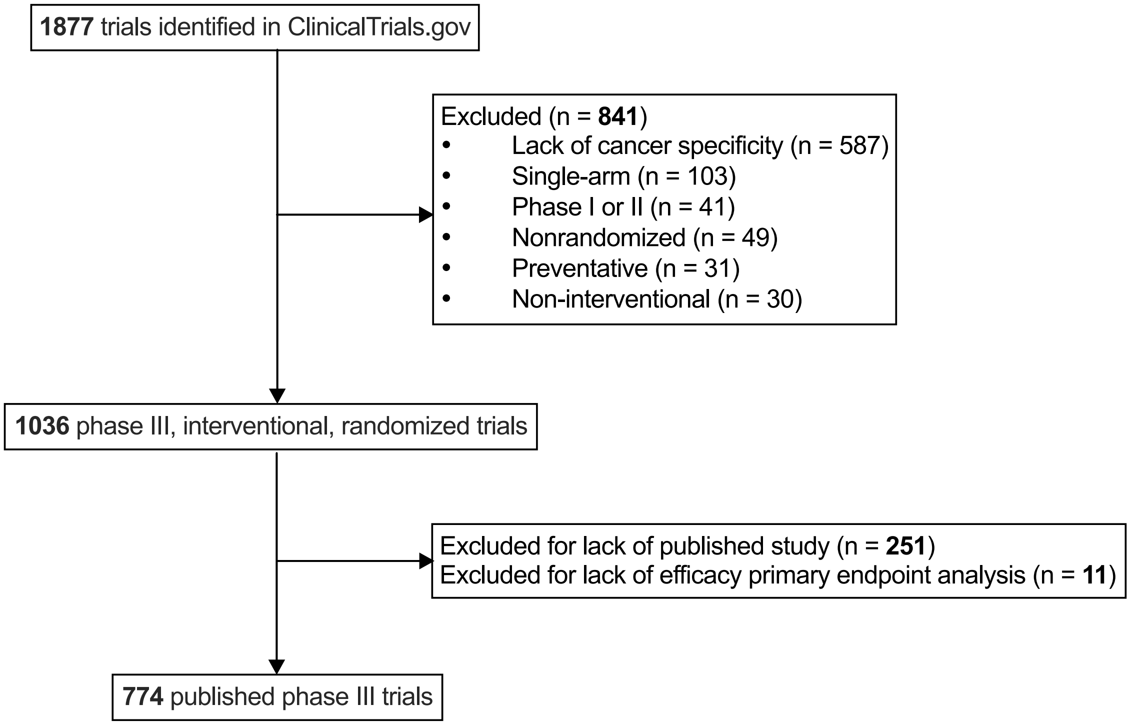

Figure S2. Directed acyclic graph used to identify the confounders for the multivariable regression. The model was developed according to the investigators' estimation of the most plausible causal relationships between variables. The outcome is highlighted in blue, which in this study is the presence of treatment-group specific inference. In this example, the predictor is highlighted in green, and the causal arrows are shown in green. The confounder is highlighted in yellow. Confounders unique to each predictor were determined by sequentially rotating the selection of the predictor in the DAGitty interface.

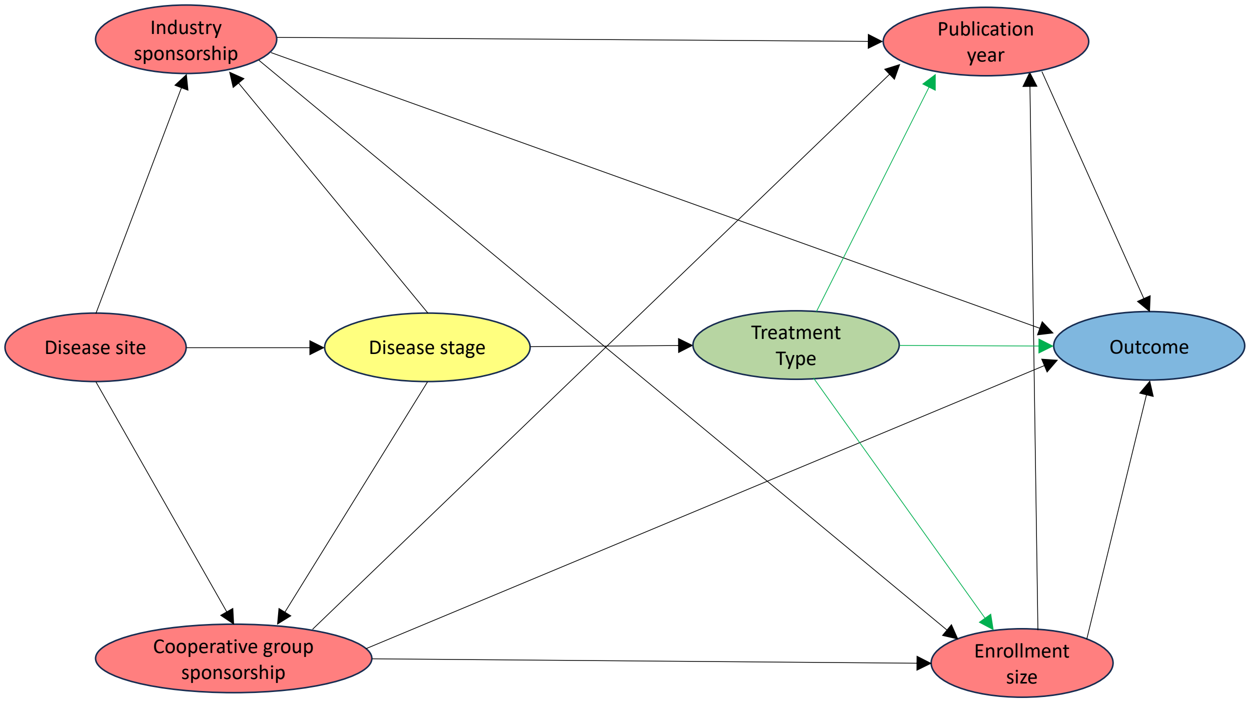

Table S1. Univariable and multivariable logistic regressions for the association of trial-level covariates with treatment-group specific inference. A separate multivariable model was used for each predictor on the basis of each predictor's unique confounders. These confounders were identified using the directed acyclic graph in Figure S2. Only the predictor estimates are shown.

Variable	OR	95% CI	*P*	aOR	95% CI	*P*
Cancer stage						
Solid M0	0.41	0.29 to 0.58	<0.0001	0.48	0.34 to 0.69	<0.0001
Solid M1	Ref			Ref		
Hematologic	0.70	0.48 to 1.03	0.07	0.99	0.63 to 1.57	0.97
Cancer type						
Breast	0.97	0.63 to 1.51	0.90	0.97	0.63 to 1.51	0.90
Gastrointestinal	2.23	1.34 to 3.80	0.003	2.23	1.34 to 3.80	0.003
Genitourinary	1.33	0.90 to 2.23	0.27	1.33	0.90 to 2.23	0.27
Hematologic	1.30	0.84 to 2.02	0.23	1.30	0.84 to 2.02	0.23
Thoracic	2.67	1.61 to 4.50	0.0002	2.67	1.61 to 4.50	0.0002
Other[a]	Ref			Ref		
Treatment type						
Systemic therapy	Ref			Ref		
Local therapy	0.68	0.31 to 1.52	0.34	0.96	0.42 to 2.22	0.92
Supportive care	0.27	0.18 to 0.40	<0.0001	0.30	0.20 to 0.46	<0.0001
Cooperative group-led	0.66	0.48 to 0.91	0.01	0.91	0.65 to 1.29	0.60
Industry-funded	2.21	1.57 to 3.11	<0.0001	1.61	1.10 to 2.34	0.01
Number of enrolled patients[b]	0.98	0.97 to 0.999	0.045	0.97	0.96 to 0.99	0.003
Publication date	1.12	1.07 to 1.17	<0.0001	1.11	1.06 to 1.17	<0.0001

Abbreviations: aOR, adjusted odds ratio; M0, nonmetastatic tumors; M1, metastatic tumors; OR, odds ratio; Ref, reference.
[a]Other cancer types: central nervous system, endocrine, gynecologic, head and neck, multiple, pediatric, sarcoma, and skin.
[b]For every additional 100 patients
